# A Novel Agent Enhances the Chemotherapeutic Efficacy of Doxorubicin in MCF-7 Breast Cancer Cells

**DOI:** 10.3389/fphar.2016.00249

**Published:** 2016-08-10

**Authors:** Liang Wang, Judy Y. Chan, Xinhua Zhou, Guozhen Cui, Zhixiang Yan, Li Wang, Ru Yan, Lijun Di, Yuqiang Wang, Maggie P. Hoi, Luchen Shan, Simon M. Lee

**Affiliations:** ^1^State Key Laboratory of Quality Research in Chinese Medicine and Institute of Chinese Medical Sciences, University of MacauMacao, China; ^2^Faculty of Health sciences, University of MacauMacao, China; ^3^Institute of New Drug Research, College of Pharmacy, Jinan UniversityGuangzhou, China

**Keywords:** danshensu, tetramethylpyrazine, doxorubicin, breast cancer, glycolysis, GRP78 protein

## Abstract

We have previously demonstrated that DT-010, a novel conjugate of danshensu (DSS) and tetramethylpyrazine (TMP), displays anti-tumor effects in breast cancer cells both *in vitro* and *in vivo*. In the present study, we investigated whether DT-010 enhances the chemotherapeutic effect of doxorubicin (Dox) in MCF-7 breast cancer cells and exerts concurrent cardioprotective benefit at the same time. Our findings showed that DT-010 was more potent than TMP, DSS, or their combination in potentiating Dox-induced toxicity in MCF-7 cells. Co-treatment with DT-010 and Dox increased apoptosis in MCF-7 cells relative to Dox alone. Further study indicated that glycolytic capacity, glycolytic reserve and lactate level of MCF-7 cells were significantly inhibited after DT-010 treatment. DT-010 also increased the expression of the pro-survival protein GRP78, which was inhibited by co-treatment with Dox. Both endoplasmic reticulum stress inhibitor 4-PBA and knockdown of the expression of GRP78 protein potentiated DT-010-mediated apoptosis in MCF-7 cells. Moreover, DT-010 inhibited Dox-induced cardiotoxicity in H9c2 myoblasts. In conclusion, DT-010 and Dox confer synergistic anti-tumor effect in MCF-7 breast cancer cells through downregulation of the glycolytic pathway and inhibition of the expression of GRP78. Meanwhile, DT-010 also protects against Dox-induced cardiotoxicity.

## Introduction

Doxorubicin, an antibiotic classified as belonging to the anthracycline group, is one of the most effective broad-spectrum anti-cancer drugs and is extensively used for the treatment of many types of cancers including breast cancer, prostate cancer, and ovarian cancer. However, many side effects, especially dose-dependent and cumulative cardiotoxicity, often limit its clinical use (Swain et al., 2003). To minimize its toxicity in heart, and multidrug resistant response in cancer cells, many strategies – such as combination with other anti-cancer or cardioprotective drugs – have been tried (Das et al., 2010; Demetri et al., 2012).

Aerobic glycolysis refers to the process of transformation of glucose in to lactate in the presence of limited oxygen. Many types of cancer cells exhibit enhanced aerobic glycolysis to meet energy demand and macromolecular biosynthesis, which partly depends on factors such as hypoxia and impairment of mitochondrial respiration (Zheng, 2012; Ganapathy-Kanniappan and Geschwind, 2013). Studies have shown that inhibition of glycolysis decreases ATP production, induces cancer cell death and increases the sensitivity of cancer cells to chemotherapeutic agents, indicating that glycoysis is a promising target for cancer therapy (Xu et al., 2005; Pelicano et al., 2006; Finley et al., 2011; Liu et al., 2014).

The endoplasmic reticulum (ER) plays a major role in promoting protein folding and maintaining cellular calcium homeostasis. Under cellular stress conditions such as oxidative stress, hypoxia and nutrient deprivation, the unfolded or misfolded proteins may accumulate in the ER, leading to ER stress (Verfaillie et al., 2013). Several studies showed ER stress is implicated in the modulation of glycolysis. ATF-4 (activating transcription factor 4), an ER stress response factor, was demonstrated as a transcriptional regulator of glycolysis (Lee et al., 2015). 2-DG (2-deoxy-D-glucose) is an inhibitor of glycolysis, which also induces ER stress (Yu and Kim, 2010). GRP78, a regulator of ER stress, is associated with aerobic glycolysis in cancer cells (Li et al., 2015). Many kinds of cancer cells exhibit increased expression of GRP78 protein in the tumor microenvironment, resulting in tumor survival, metastasis and resistance to chemotherapy (Lee, 2007). Thus, inhibition of GRP78 is a strategy to overcome chemotherapeutic resistance and improve cancer therapy.

Our previous findings indicated that a DSS and tetramethylpyrazine (TMP) derivative, DT-010, was more potent than their parental compounds or their combination in inhibiting proliferation of breast cancer cells both *in vitro* and *in vivo* by targeting mitochondrial complex II (Wang et al., 2016). In this study, we will investigate whether DT-010 enhances the chemotherapeutic effect of Dox in MCF-7 breast cancer cells and its underlying mechanisms. Furthermore, the cardioprotective effects of DT-010 against Dox-induced cardiotoxicity will also be determined.

## Materials and Methods

### Materials

**FIGURE 1:**
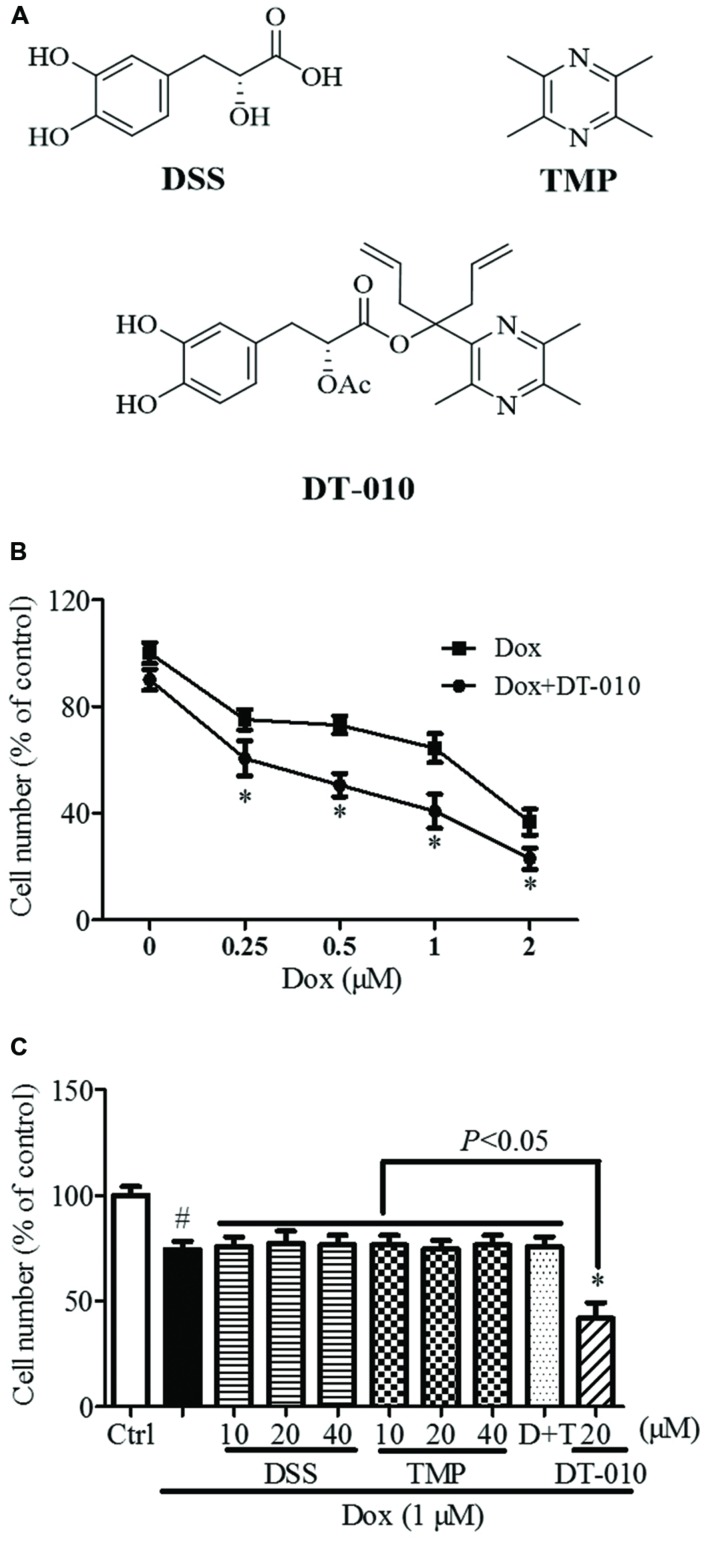
**Synergistic anti-tumor effect of DT-010 and Dox on MCF-7 breast tumor cells. (A)** Chemical structures of DSS, TMP, and DT-010 (Wang et al., 2016). **(B)** Cell numbers of MCF-7 cells were determined after DT-010 and different concentrations of Dox treatment. **(C)** MCF-7 cells were treated with different concentration of DSS, TMP, D+T (DSS+TMP), DT-010 and Dox (1 μM) for 24 h, and cell numbers were then counted. *^#^P* < 0.05 vs. Ctrl and *^∗^P* < 0.05 vs. Dox.

DT-010 (purity > 98%, **Figure 1A**) were synthesized at Jinan University, China (Scheme 1). TMP and DSS (**Figure 1A**) were purchased from Shanghai Banghai Chemical Company (China) and Xi’an Honson Biotechnology (China), respectively. The Dulbecco’s Modified Eagle’s Medium (DMEM), penicillin-streptomycin and fetal bovine serum (FBS) used in this study were obtained from Life Technologies (USA). 3-(4, 5-dimethylthiazol-2-yl)-2, 5-diphenyltetrazoliumbromide (MTT) was purchased from Sigma-Aldrich (USA). GRP78 siRNA was provided by Santa Cruz Biotechnology (USA). An XF Glycolysis Stress Test Kit was purchased from Seahorse Bioscience (USA). Lactate assay kit was purchased from BioVision (USA).

Scheme 1 Synthesis of DT-010. Reagents and conditions: (a) KMnO_4_, 45°C, overnight, 50%; (b) CH_3_CH_2_OH, EDCI, DMAP, r.t., 84%; (c) C_3_H_5_MgBr, THF, 0°C to r.t., 34%; (d) Ac_2_O, HClO_4_, r.t., 3 h, 36%; (e) C_2_Cl_2_O_2_, DMF, CH_2_Cl_2_; (f) n-C_4_H_9_Li, THF, 0°C to r.t., 41%; (g) Na_2_CO_3_, CH_3_OH, H_2_O, 82%.

### Cell Culture

MCF-7 and H9c2 cells were cultured in DMEM medium with 10% FBS at 37°C in an incubator containing 5% CO_2_ and 95% air. Cells were used until they reached 70–80% confluence.

### Measurement of Cell Viability, Cell Numbers, and Apoptosis

Cell viability was assessed by MTT assay. Briefly, H9c2 cells were cultured in 96 well plates for 24 h. After 24 h of treatment with Dox in the absence or presence of DT-010, cells were incubated in medium containing 1 mg/ml MTT. The formazen was then dissolved with 100 μl DMSO after incubation at 37°C. SpectraMax M5 Microplate Reader was used to detect the absorbance at 570 nm. Cell numbers were determined as described previously (Wang et al., 2015). Briefly, MCF-7 cells were cultured in 96-well plates for 24 h. After treatment with DT-010 and DT-010 for 24 h, cells were stained with DAPI and then visualized by the In Cell Analyzer 2000 system. The number of cells was calculated using Developer Toolbox software. For apoptosis assay, MCF-7 cells were stained with Hoechst 33342 for 15 min to measure apoptotic cells. The apoptotic cells with changes in chromatin condensation were analyzed by the In Cell Analyzer 2000 system.

### Determination of Metabolic Parameters

MCF-7 cells at a density of 1 × 10^4^ cells/well were cultured in 24-well tissue microplates (Seahorse) for 24 h (Each group has at least three wells). After 12 h of DT-010 treatment, the medium was then replaced with Seahorse base medium (pH = 7.4) and placed the plate into 37°C non-CO_2_ incubator for 1 h. The ECAR and OCR values were measured before and after the injection of metabolic reagents from the XF Glycolyis Stress Test Kit.

### Evaluation of Lactate Level

MCF-7 cells were plated in 96-well plates at a density of 6 × 10^3^ cells/well. After 12 h of DT-010 treatment, the production of lactate in culture medium was measured using Lactate assay kit (BioVision) and normalized to the number of cells.

### Western Blot Analysis

Cells lysate was extracted as previously report (Wang et al., 2015). Briefly, MCF-7 cells were washed with PBS and lysed by cell lysis buffer supplemented with 1% phenylmethanesulfonyl fluoride and 1% cocktail. After 30 min of incubation on ice, the samples were centrifuged (12,000 × *g*) for 20 min at 4°C. The concentration of supernatant proteins was measured according to the protocol for BCA protein assay (Thermo Scientific). After being denatured at 95°C for 5 min, proteins were then separated by sodium dodecyl sulfate polyacrylamide gel electrophoresis (SDS-PAGD) and transferred to polyvinylidene fluoride membrane using semi-dry protein transfer. The membrane was block by 5% milk for 1 h and incubated with primary antibodies against β-actin, cleaved-PARP, p53 (Cell Signaling) and GRP78 (Santa Cruz Biotechnology). After incubation at 4°C overnight, the membrane was incubated with secondary antibodies (Cell Signaling) for 1 h at room temperature. Finally, the advanced enhanced ECL system (GE Healthcare) was used to detect the immune complexes and the bolts were quantified by Quantity One software (Bio-Rad).

### Statistical Analysis

Data are shown as mean ± standard deviation (SD). Differences among the groups were compared by one-way ANOVA followed by Turkey’s multiple comparison tests. *P* < 0.05 was considered as statistically significant.

## Results

### DT-010 and Dox Display Synergistic Anti-tumor Effects against MCF-7 Breast Tumor Cells

As shown in **Figure 1B**, the numbers of MCF-7 cells were significantly decreased after 24 h of Dox (1 μM) treatment, and were further reduced after DT-010 treatment. Co-treatment with DT-010 and Dox was more potent than DSS, TMP, DSS+TMP, and Dox combination in inducing cell death of MCF-7 cells (**Figure 1C**).

### DT-010 Increases Dox-Induced Apoptosis of MCF-7 Cells

**FIGURE 2:**
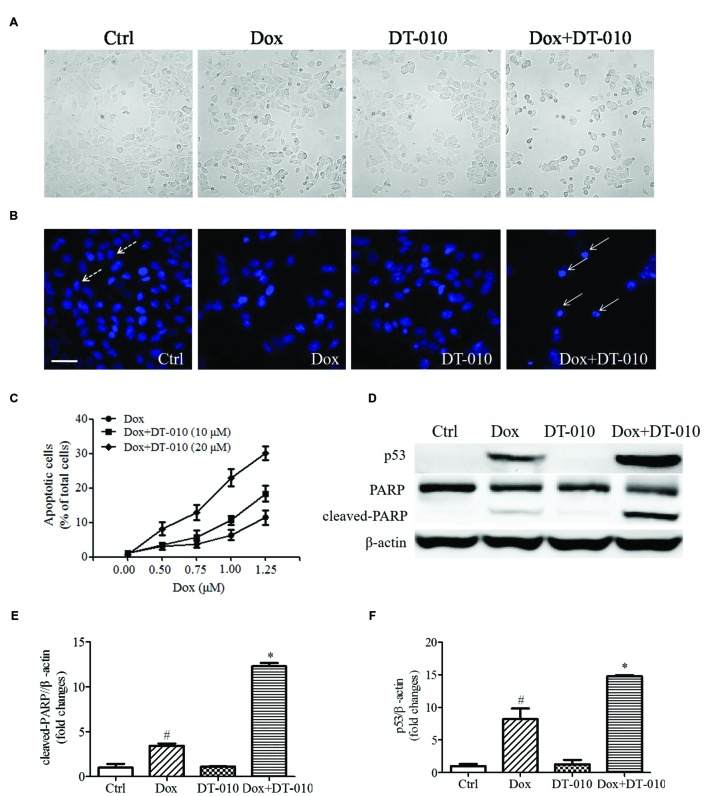
**DT-010 enhanced Dox induced apoptosis in MCF-7 cells. (A)** Representative images of the cell morphology of MCF-7 cells. **(B)** Representative images of apoptotic cells in MCF-7 cells, which were stained with Hoechst33342. Dotted and solid arrows indicate the normal cells and apoptotic cells with chromatin condensation, respectively. Scale bar: 50 μm. **(C)** The percentage of apoptotic cells in MCF-7 cells after 24 h of DT-010 and Dox treatment. **(D)** The expression of p53, PARP and cleaved-PARP proteins in MCF-7 cells were determined by Western blot after 24 h of Dox treatment in the presence or absence of DT-010. **(E,F)** Densitometric quantification of the expression of cleaved-PARP and p53 protein.*^#^P* < 0.05 vs. Ctrl and ^∗^*P* < 0.05 vs. Dox.

To investigate whether DT-010 increases Dox-induced apoptosis in MCF-7 cells. MCF-7 cells were co-treated with DT-010 and Dox for 24 h. **Figure 2A** shows that co-treatment with Dox and DT-010 for 24 h changes cell morphology as compared with Dox treated group. Dox treatment alone induced apoptosis in MCF-7 cells, and the combination of DT-010 and Dox further increased cell apoptosis (**Figures 2B,C**). This is consistent with the data showing that the expression of apoptosis-related proteins p53 and cleaved-PARP increased after Dox treatment (**Figures 2D–F**), which was further amplified after DT-010 and Dox co-treatment.

### DT-010 Blocks Glycolytic Pathway in MCF-7 Cells

**FIGURE 3:**
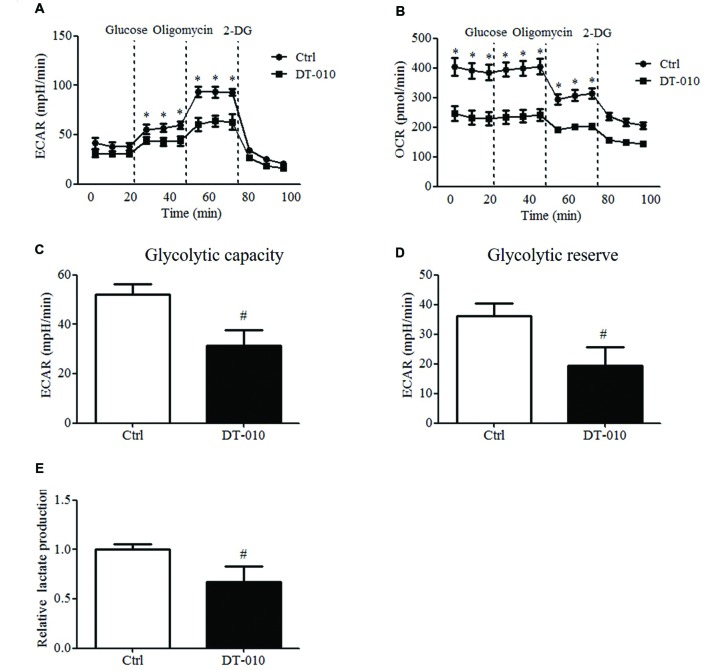
**DT-010 blocked glycolytic pathway of MCF-7 cells.** MCF-7 cells were treated with DT-010 for 12 h, and the ECAR **(A)** and OCR **(B)** were measured with XF24 extracellular flux analyzer. *^∗^P* < 0.05 vs. DT-010 treated group. **(C,D)** The glycolytic capacity and glycolytic reserve in MCF-7 cells were calculated. *^#^P* < 0.05 vs. Ctrl. **(E)** MCF-7 cells were treated with DT-010 for 12 h. Levels of lactate were measured according to lactate assay kit. *^#^P* < 0.05 vs. Ctrl.

The effects of DT-010 on the glycolysis of MCF-7 cells were investigated. The changes of ECAR and OCR were monitored by the Seahorse XF Extracellular Flux Analyzer. As shown in **Figures 3A,C,D**, the glycolytic capacity and glycolytic reserve significantly decreased after DT-010 treatment. DT-010 also inhibits the basic level of OCR in MCF-7 cells (**Figure 3B**), which is consistent with our previous study, showing that DT-010 inhibits mitochondrial respiration in breast cancer cells. Further study showed DT-010 treatment inhibited the level of lactate in culture medium (**Figure 3E**).

### Dox Inhibits DT-010-Induced GRP78 Expression in MCF-7 Cells

**FIGURE 4:**
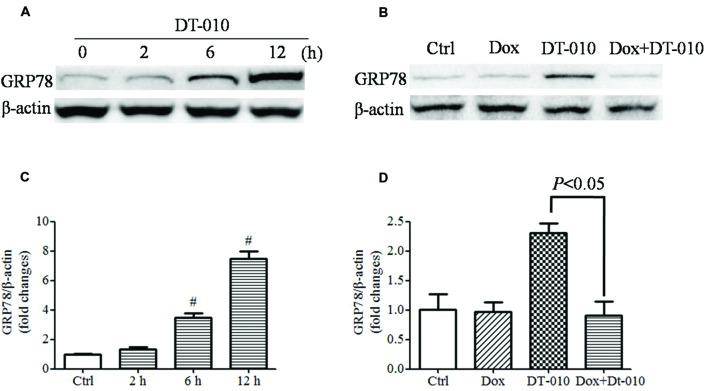
**Dox inhibited DT-010-induced GRP78 expression in MCF-7 cells.** Immunoblots **(A)** and densitometric analysis **(C)** of the expression of GRP78 protein were determined after DT-010 treatment in MCF-7 cells. **(B)** MCF-7 cells were treated with DT-010 for 24 h in the presence or absence of Dox. Representative immunoblots for the expression of GRP78 and β-actin proteins were exhibited. **(D)** Densitometric analysis showed the expression of GRP78 protein. *^#^P* < 0.05 vs. Ctrl.

The effects of DT-010 on GRP78 expression in MCF-7 cells were determined. Images of immunoblots in **Figure 4A** and densitometric analysis in **Figure 4C** showed that DT-010 increased the expression of GRP78 protein in a time dependent manner. Previous study showed that Dox prevented ER stress inducer-mediated GRP78 expression (Kim et al., 2006). In this study, we examined the effect of Dox on DT-010-induced GRP78 expression in MCF-7 cells. DT-010 treatment promoted the expression of GRP78 protein, which was inhibited by Dox and DT-010 co-treatment (**Figures 4B,D**).

### Inhibition of GRP78 Expression Enhances DT-010-Induced Cell Apoptosis

**FIGURE 5:**
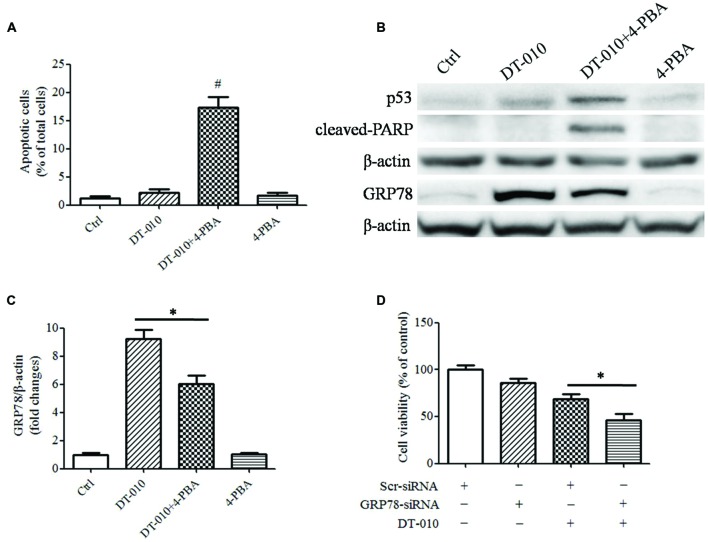
**Inhibition of GRP78 expression enhanced DT-010-induced cell apoptosis in MCF-7 cells. (A)** The percentage of apoptotic cells in MCF-7 cells was recorded after 24 h of DT-010 treatment in the presence or absence of ER stress inhibitor 4-PBA. *^#^P* < 0.05 vs. DT-010 treated group. **(B)** MCF-7 cells were treated with Dox and DT-010 for 24 h, the expression of p53, cleaved-PARP, β-actin proteins were measured by western blot. The expression of GRP78 protein was determined after 12 h of Dox and DT-010 treatment. Representative immunoblots for the expression of p53, cleaved-PARP, β-actin and GRP78 proteins. **(C)** Densitometric quantification of the expression of GRP78 protein. **(D)** GRP78 knockdown further promotes DT-010-induced cell death in MCF-7 cells. ^∗^ means significant difference between treatments (*P* < 0.05).

To determine whether there were synergistic anti-tumor effects of DT-010 in combination with Dox via the inhibition of GRP78 expression, MCF-7 cells were co-treated with DT-010 and the ER stress suppressor, 4-PBA. DT-010 and 4-PBA co-treatment markedly increased apoptosis in MCF-7 cells as compared with DT-010 or 4-PBA treated alone (**Figure 5A**). Similarly, 4-PBA potentiated DT-010-induced expression of the apoptosis-related proteins p53 and cleaved-PARP, and suppressed DT-010-induced expression of GRP78 protein (**Figures 5B,C**). Moreover, GRP78 knockdown using siRNA significantly reduced cell viability after DT-010 treatment relative to DT-010 alone (**Figure 5D**).

### DT-010 Protects against Dox-Induced Cardiotoxicity in H9c2 Cells

**FIGURE 6:**
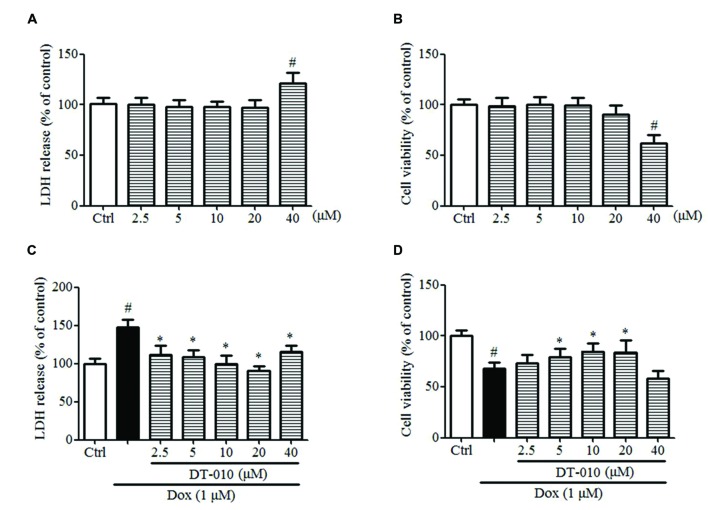
**DT-010 protected against Dox-induced toxicity in H9c2 cells. (A,B)** H9c2 cells were treated with different concentrations of DT-010 for 24 h and the toxicity of DT-010 in H9c2 cells was measured by LDH assay and MTT assay, respectively. Dox treatment for 24 h significantly increased the cytotoxicity **(C)** and decreased cell viability **(D)** in H9c2 cells, which were reversed by DT-010 co-treatment. ^#^*P* < 0.05 vs. Ctrl and ^∗^*P* < 0.05 vs. Dox.

The effects of DT-010 on Dox-induced toxicity were investigated in this study. **Figures 6A,B** indicate that DT-010 exposure alone showed no toxicity at concentrations below 20 μM as detected by LDH assay and MTT assay. Further study indicates Dox causes cytotoxicity and decreases cell viability in H9c2 cells, while DT-010 co-treatment reduces LDH release (**Figure 6C**) and preserves cell viability (**Figure 6D**) in a dose-dependent manner as compared with the DT-010 treated group. These data suggest that DT-010 inhibits Dox-mediated cardiotoxicity in H9c2 cells.

## Discussion

We have shown that DT-010, a derivative of danshensu (DSS) and TM), exhibits anti-tumor effects in breast cancer cells via the inhibition of mitochondrial complex II, resulting in ROS generation and mitochondrial dysfunction (Wang et al., 2016). DSS is a major ingredient of Danshen, which is one of the most commonly used Chinese herbs for the treatment of cardiovascular disease (Cui et al., 2013; Yin et al., 2013; Yu et al., 2014). TMP, a major alkaloid ingredient of the Chinese herb Chuanxiong, shows pharmacological effects such as anti-oxidation, anti-apoptosis, and protection against ischemic brain injury (Liao et al., 2004; Juan et al., 2007; Sue et al., 2009). On the other hand, TMP also displays anti-cancer effects and overcomes Dox-resistance in MCF-7 breast cancer cells (Zhang et al., 2012; Yi et al., 2013). Based on previous study, we further evaluated the potential synergistic anti-tumor effects of DT-010 with Dox in MCF-7 cells. We found that DT-010 and Dox co-treatment displayed much better anti-tumor effects than its parental compounds, separately or in combination, in the presence of Dox. Further study showed that Dox induced apoptosis in MCF-7 cells, which was further increased after DT-010 co-treatment.

A growing body of evidence shows that inhibition of glycolysis may kill cancer cells, overcome multidrug resistance and enhancing the efficacy of chemotherapeutic agents, thereby suggesting that glycolysis is a novel target for cancer therapy. Glycolysis inhibitors such as 2-deoxyglocose, 3-bromopyruvate, and lonidamine decrease ATP levels in cancer cells and display anti-cancer effects both *in vitro* and *in vivo* (Pelicano et al., 2006). Overexpression of SIRT3 has been shown to inhibit tumor proliferation through the repression of glycolysis (Finley et al., 2011). 2-DG increases the chemotherapeutic efficacy of Dox and paclitaxel in cancer cells *in vivo* (Maschek et al., 2004). Inhibition of glycolysis also enhances the sensitivity of multidrug-resistant cells to anti-cancer agents (Xu et al., 2005). Our data showed that DT-010 inhibited glycolytic capacity, glycolytic reserve and lactate level of MCF-7 cells, improving the anti-cancer effects of Dox in MCF-7 cells, which is consistent with prior studies showing inhibition of glycolysis as a relevant strategy for improving effects of conventional chemotherapeutic drugs such as Dox.

GRP78 confers chemotherapeutic resistance in a variety of cancers. Studies showed that suppression of GRP78 enhanced the anti-cancer effects of etoposide in NCI-H446 cancer cells (Wang et al., 2008). The anti-cancer effects of etoposide and doxorubicin in CHO cells were inhibited after GRP78 overexpression (Reddy et al., 2003). Moreover, overexpression of GRP78 reduced cells apoptosis in response to etoposide and cisplatin in glioma cells, while the down-regulation of GRP78 increased the sensitivity of glioma cells to etoposide and cisplatin (Lee et al., 2008). In the present study, we observed that the expression of GRP78 protein was elevated after DT-010 treatment in MCF-7 cells. Previous study showed that Dox inhibited the expression of GRP78 protein and ATF-4 mRNA after treatment with the ER stress inducer thapsigargin in MCF-7 cells (Kim et al., 2006). We then provided evidence that the ER stress inhibitor, 4-PBA, decreased the expression of GRP78 protein and increased apoptosis in MCF-7 cells after DT-010 treatment. Furthermore, knockdown of GRP78 using siRNA potentiated DT-010-induced cell death in MCF-7 cells. These results indicated that DT-010 and Dox displayed synergistic anti-tumor effects partly via GRP78 downregulation.

In this study, we also investigated the cardioprotective effects of DT-010 on Dox-induced cardiotoxicity. The data showed that Dox triggered toxicity in H9c2 cells, which was significantly inhibited by DT-010 co-treatment.

## Conclusion

This study demonstrated that DT-010 and Dox displayed a synergistic anti-tumor effect in breast cancer cells. Further study indicated that the anti-tumor effect of DT-010 and Dox was correlated with the inhibition of glycolysis and GRP78-mediated pro-survival pathway. Moreover, DT-010 protected against Dox-mediated cardiotoxicity in H9c2 cells. This study suggested the potential utility of the DT-010 and Dox combination for improving the anti-tumor efficacy of Dox in MCF-7 breast cancer cells.

## Author Contributions

LiaW, GC, JC, and XZ drafted manuscript drafting. LiaW and ZY carried out experiments. LW, LD, RY, and MHprovided technical advice. YW was responsible for chemical design and synthesis. LS and SL were responsible for planning and monitoring the whole project.

## Conflict of Interest Statement

The authors declare that the research was conducted in the absence of any commercial or financial relationships that could be construed as a potential conflict of interest.
